# Reactionary Bone Changes in Long-Standing Pyogenic Granuloma: A Case Report

**DOI:** 10.7759/cureus.53021

**Published:** 2024-01-26

**Authors:** Rosalyn Lalremtluangi, Suwarna Dangore-Khasbage

**Affiliations:** 1 Oral Medicine and Radiology, Sharad Pawar Dental College and Hospital, Datta Meghe Institute of Higher Education and Research, Wardha, IND

**Keywords:** vascular, trabeculae, pyogenic granuloma, osteogenesis, exophytic

## Abstract

Pyogenic granuloma is a reactive lesion that is commonly seen in the skin and oral cavity. Though it is an unfortunate misnomer, being neither pyogenic nor a true granuloma, the name has been used for years. In the oral cavity, it presents as a growth mostly situated on the gingiva, but may also occur on the tongue, buccal and labial mucosa, and palate, and may even be seen in relation to dental implants. The lesion is usually bright or purple red in color, soft in consistency, relatively painless, and appears highly vascularized. Local etiologic factors are usually poor oral hygiene or chronic irritation. Histopathologically it is an inflammatory hyperplasia of the connective tissue with exuberant vascularity. Sometimes, this lesion may present with unusual histopathological patterns, which could lead to difficulty in diagnosis. Long-standing pyogenic granuloma may show histologic changes such as localized osteogenesis in the matrix of the lesion which could potentially lead to complications in the area of the lesion. This article reports a rare case with rare histopathological features in an 18-year-old female presenting with long-standing soft tissue gingival growth.

## Introduction

Pyogenic granuloma (PG), sometimes referred to as granuloma pyogenicum, refers to a commonly acquired, vascular tumor (benign) that arises in tissues such as the mucous membranes and skin [1]. More accurately, it is referred to as lobular capillary hemangioma. The name PG is believed to be a misnomer since the lesion is not accompanied by pus accumulation [2]. The etiology of PG is clearly unknown, but because PG can regress when initiating stimuli are removed, it may qualify as a vascular hyperplasia. It is considered that PG is a “reactive” or “reparative” tumor process that embodies an exuberant connective tissue proliferation. Possible predisposing factors may include chronic irritation, trauma, increased hormone levels in females, infections, viral oncogenes, and microscopic arteriovenous anastomoses [3,4].

Histopathologically, PG is characterized by exophytic to polypoid proliferation of capillary-sized blood vessels, circumscribed and in a lobular arrangement [5]. When PG is chronic, the characteristic vascular content may be associated with trabeculae and bone formation which may transform into peripheral ossifying fibroma [6]. This is a case presentation of an 18-year-old female with PG presenting unique histologic features with a discussion of the possible differential diagnosis.

## Case presentation

**Figure 1:**
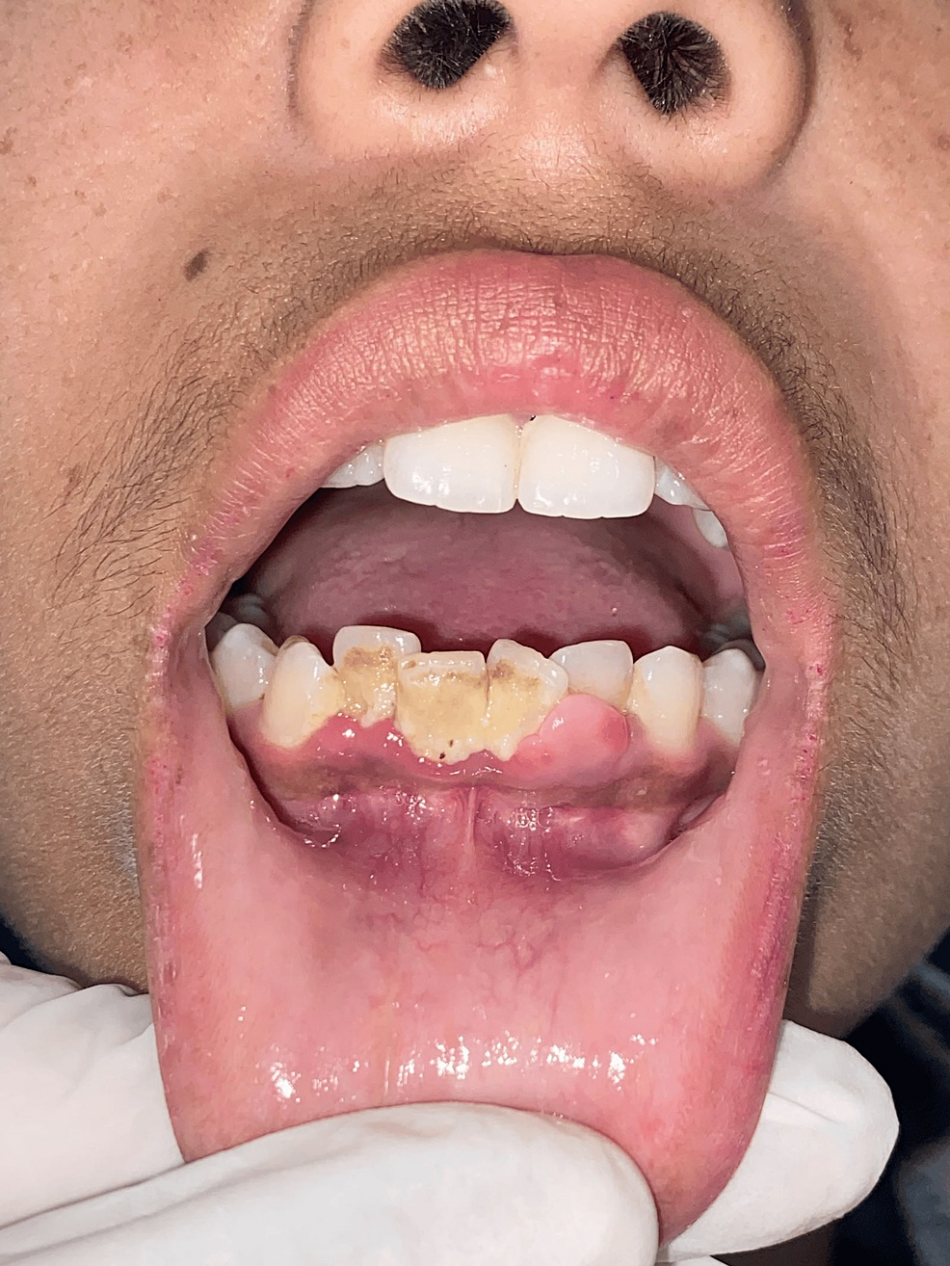
Localized soft tissue growth with 31, 32 region

**Figure 2:**
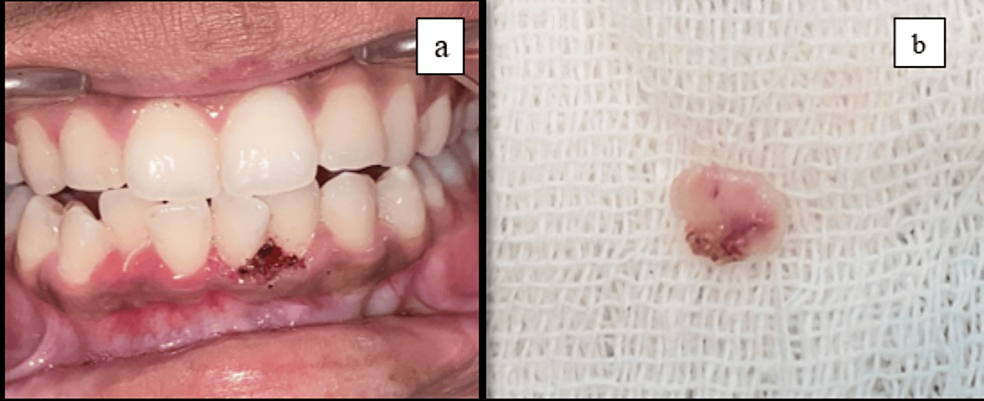
(a) Excision done on the soft tissue growth. (b) Excised tissue.

**Figure 3:**
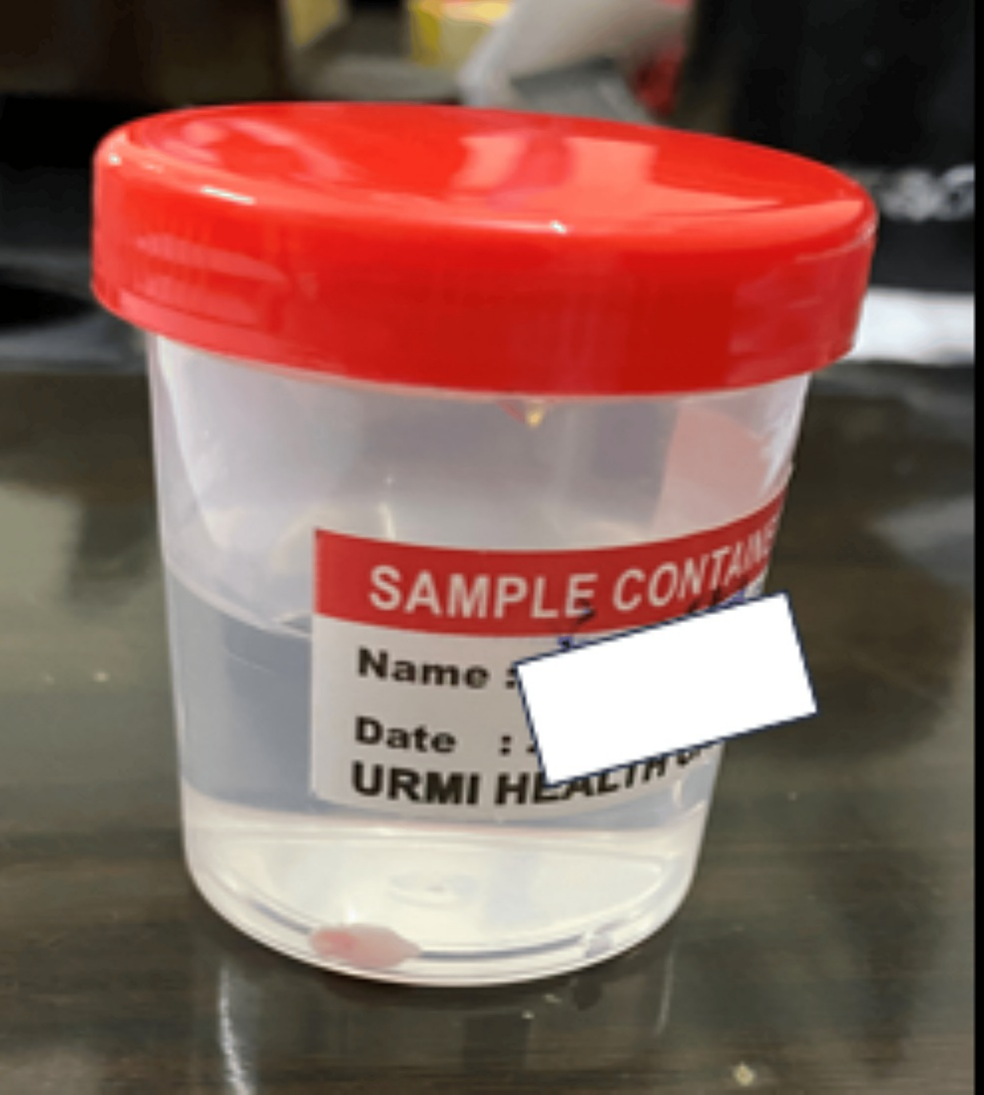
Excised tissue placed in formalin solution.

**Figure 4:**
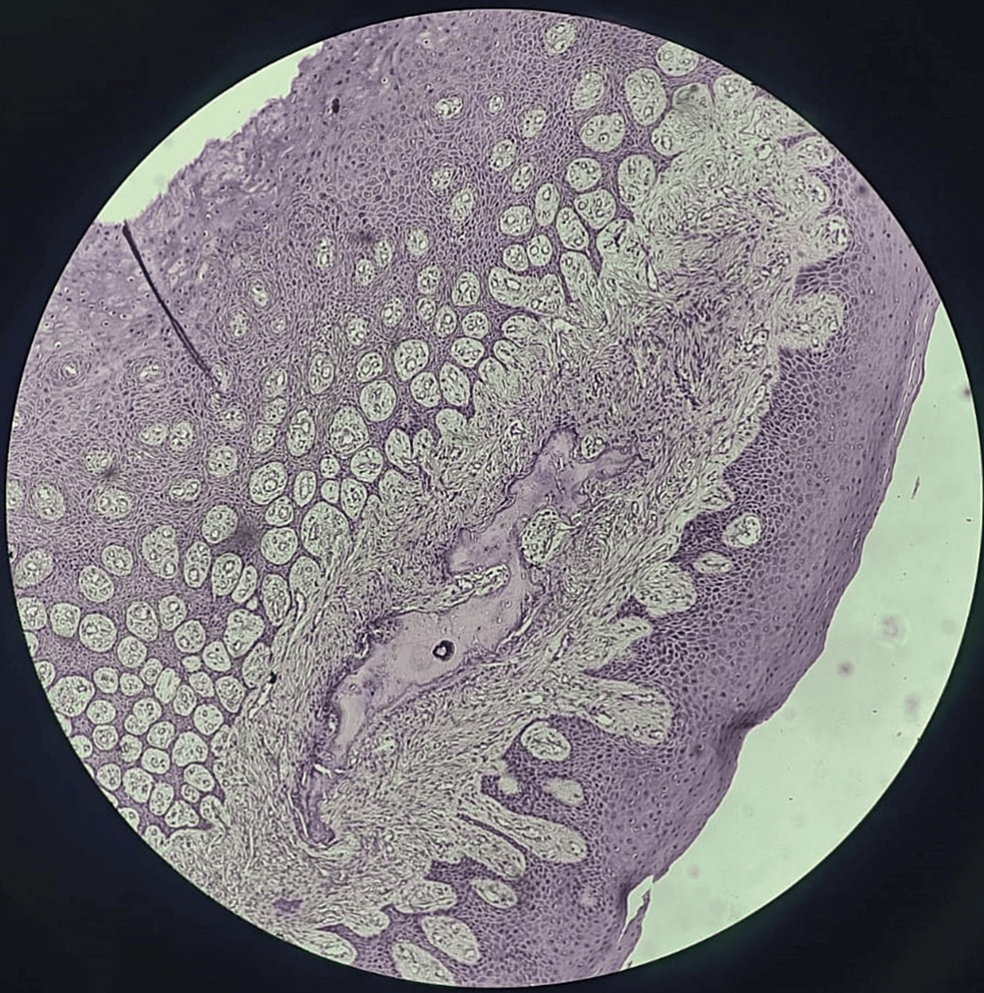
Overlying hyperplastic epithelium and underlying connective tissue stroma comprising numerous blood capillaries and dense inflammatory cell infiltrate with bony trabeculae.

An 18-year-old-female came to the Department of Oral Medicine and Radiology at Sharad Pawar Dental College and Hospital, Datta Meghe Institute of Higher Education and Research (DMIHER) with a chief complaint of soft tissue growth in the lower anterior tooth region for one year. The patient reported that the growth was initially small and then gradually progressed to the current size and is painless. The patient also gave a history of bleeding from the area while brushing teeth. There was no difficulty in speech or mastication. On examination, a single exophytic growth was seen on the marginal gingiva of the lower left incisors' region with a size of 0.5 x 0.5 cm approx., roughly oval in shape, pale pink in color with a smooth surface which is soft in consistency and non-tender on palpation (Figure 1). No bleeding or pus discharge during palpation of the lesion. There was mild crowding seen in the mandibular anterior with a chunk of calculus in the vicinity of the growth which appears to be the local irritating factor for the growth. There was no mobility on the adjacent tooth. Taking into consideration the possible causative factor, location, and appearance of the growth, clinical diagnosis was given as PG while peripheral giant cell granuloma, peripheral ossifying fibroma, and fibroma were considered in the differential diagnosis. In an attempt to remove the causative factor, scaling of teeth was carried out on priority on the same day. The patient was recalled after seven days, and it was observed that the size of the growth has not diminished. So, the excision of the growth was done with a diode laser (Figures 2a, 2b). The excised tissue specimen was kept in formalin solution and was sent for Histopathological evaluation (Figure 3). The histopathological report revealed overlying hyperplastic epithelium and underlying stroma of connective tissue comprising numerous blood capillaries and dense inflammatory cell infiltrate along with osteoid formation and bony trabeculae. All these features were suggestive of PG with reactive bone formation (Figure 4). The patient was recalled after seven days where healing of the surgical site was evident. The patient is on regular follow-up where satisfactory result is seen without any recurrence of the growth.

## Discussion

PG of the oral cavity is seen most commonly on the gingiva, particularly the marginal gingiva, and is considered to be around 75% of all cases. It may also occur on the lips, tongue, buccal mucosa, and very rarely on other areas [7]. The lesion is typically an elevated, sessile, pedunculated, or smooth vascular mass with a smooth, lobulated, or even warty surface. It is frequently ulcerated and has a propensity to bleed either on its own or in response to mild trauma [8]. These lesions can affect both sides of the gingiva, including the interdental papilla; however, they most frequently appear in the labial aspect as opposed to the palatal/lingual aspects. It could be unilateral or bilateral, single, or occur at multiple sites. It is deep red or red purple, depending upon its vascularity, painless, and rather soft in consistency. Certain lesions may exhibit a brown cast if there has been tissue mass hemorrhage [9,10].

PG may grow rapidly, reach its full size, and then remain static for a long period of time. Initially, the lesion appears to be bright red in color due to the underlying vascularity of the lesion. But when the growth remains stagnant for a long period of time PG may undergo organization and healing, which may be evident in histological examination with features of decreased vascularity, decreased inflammation, and fibroblast proliferation alongside the capillary network causing constriction of the capillary network which may lead to the growth appearing from bright red to pale pink due to maturation of fibroblast [11,12]. Furthermore, local osteoid formation may occur on the matrix of the lesion which can lead to the formation of bony trabeculae leading to reactive bone formation as seen in this case report which may further convert the growth to peripheral ossifying fibroma which could possibly cause more complications around the area of the lesion [13].

For this particular case, differential diagnoses taken into consideration were fibroma, peripheral giant cell granuloma, and peripheral ossifying fibroma and along with this, hormonal tumor, epulus fissuratum, and parulis may also be added as they present more or less similar features clinically. Differentiating features may be decided according to the etiology of the lesion or histopathological examination. Recurrence of the lesion is quite low [14]. More research may be required on the pathogenesis of these reactive lesions which could shed light on this continuum concept.

Management of the PG is surgical excision. Large lesions may require confirmation by incision biopsy before excision. Small lesions can be treated by removal of causative factors along with observation. Alternative methods may include the laser (Nd: YAG, CO2), cryosurgery, STS (sodium tetradecyl sulfate) sclerotherapy, and electrodessication. In the present case also, we have performed excision of the lesion and removal of causative factors. Lesions in women who are pregnant need to be managed with utmost care as the risk of recurrence and bleeding may lead to complications such as eclampsia [15].

## Conclusions

This case report supports the change of PG where reactive bone formation is observed which could possibly lead to peripheral ossifying fibroma as the lesion was chronic and long-standing. This reactive bone formation is believed to be due to stimulation of the adjacent bone where osseous cells migrate through periodontal tissue to the affected site. After careful examination of the soft tissue growth, immediate intervention was done by excising the lesion where a satisfactory result was achieved without any recurrence of the lesion.
